# Les complications du diverticule de Meckel chez l'enfant: à propos de 18 cas

**DOI:** 10.11604/pamj.2019.33.113.18756

**Published:** 2019-06-14

**Authors:** Mohammed Tazi Charki, Mohammed-Amine Oukhouya, Zineb Benmassaoud, Abdelhalim Mahmoudi, Khalid Khattala, Youssef Bouabdallah

**Affiliations:** 1 Centre Hospitalier Universitaire Hassan II, Université Sidi Mohamed Ben Abdellah, Département de Chirurgie Pédiatrique, Fès, Maroc

**Keywords:** Diverticule de Meckel, complications, occlusion intestinale, hémorragie digestive, nouveau-né, Meckel's diverticulum, complications, intestinal occlusion, digestive hemorrhage, newborn

## Abstract

Le diverticule de Meckel (DM) est une persistance du canal omphalo-mésentérique. Il est souvent asymptomatique, mais peut être responsable de complications diverses avec des tableaux cliniques variables notamment chez l'enfant. Nous avons réalisé une étude rétrospective au Service de Chirurgie Pédiatrique du CHU Hassan II de Fès (Maroc), sur les DM compliqués chez l'enfant dans le but de décrire les aspects cliniques, radiologiques et thérapeutiques. Sur une période de 10 ans (janvier 2009- décembre 2018), 18 enfants (15 garçons et 3 filles) âgés de 1 jour à 15 ans (âge moyen de 5 ans) ont été opérés pour une complication du DM. L'invagination intestinale aigue et l'occlusion intestinale étaient les complications les plus fréquentes. Les autres complications décrites sont l'infection du DM (1 cas) et l'hémorragie digestive (2 cas). Deux formes néonatales rares ont été décrites (occlusion néonatale et fistule sur omphalocèle). L'imagerie basée sur la radiographie de l'abdomen sans préparation (ASP), l'échographie et la tomodensitométrie (TDM) abdominale n'ont objectivé le DM en aucun cas. La scintigraphie a été réalisée pour les 2 cas ayant une rectorragie et elle a permis de faire le diagnostic du DM dans un cas. Trois patients ont été opérés par laparoscopie avec résection du DM avec anastomose intestinale par out-laparoscopie. Les autres patients ont été opérés par laparotomie. L'iléostomie a été réalisée dans un cas suivi d'un rétablissement secondaire. L'évolution était bonne pour tous les patients hormis un cas de lâchage d'anastomose. Sur le plan anatomo-pathologique, deux cas d'hétérotopie ont été notés.

## Abstract (en)

Meckel’s diverticulum (MD) is a remnant of omphalomesenteric channel. It is often asymptomatic but it can be responsible for various clinical complications and variable clinical status especially in children. We conducted a retrospective study on complications of MD among children hospitalized in the division of Paediatric Surgery at the University Hospital Hassan II, Fez, Morocco. The study aimed to describe the clinical, radiological and therapeutic features of MD. The study was conducted over a period of 10 years (January 2009 - December 2018) and involved 18 children (15 boys and 3 girls) aged 1 day - 15 years (with an average age of 5 years) who had undergone surgery for complications of MD. Acute intussusception and intestinal occlusion were the most frequent complications. Other complications included: infection of the MD (1 case) and digestive hemorrhage (2 cases). Two rare types of neonatal Meckel’s diverticulum were described (neonatal occlusion and fistula associated with omphalocele). In no case, abdominal X-ray without treatment, ultrasound and CT scan showed MD. Scintigraphy was performed in 2 patients with hematochezia and it helped to make the diagnosis of MD in one case. Three patients underwent laparoscopic surgery with resection of the MD and intestinal anastomosis with laparoscopy. The other patients underwent laparotomy. Ileostomy was performed in one case, followed by secondary recovery. Patient’s outcome was good, except for one case of anastomotic leakage. Anatomopathological examination showed two cases of heterotopia.

## Introduction

Décrit pour la première fois en 1809 par Johann Friedrich Meckel, le diverticule de Meckel (DM) est une persistance du canal omphalo-mésentérique. Il représente la malformation congénitale la plus fréquente du tube digestif [1]. Le DM est généralement latent et de découverte fortuite, mais peut être responsable de complications diverses avec des tableaux cliniques variables notamment chez l'enfant [2]. Les complications sont essentiellement obstructives, hémorragiques ou infectieuses, mais d'autres complications plus rares ont été rapportées [1, 3-5]. La spécificité de l'imagerie reste faible et le diagnostic se fait souvent en per-opératoire pour une indication non exclusivement guidée par la suspicion du DM [3, 6, 7]. Le but de cette étude est de décrire les aspects cliniques, radiologiques et thérapeutiques des diverticules de Meckel compliqués chez l'enfant.

## Méthodes

Il s'agit d'une étude rétrospective menée sur 10 ans (janvier 2009-décembre 2018) portant sur les cas de DM compliqués pris en charge au Service de Chirurgie Pédiatrique du Centre Hospitalo-universitaire Hassan II de Fès (Maroc). Les DM non compliqués de découverte fortuite lors d'une exploration abdominale ont été exclus de cette étude. Les paramètres épidémiologiques, cliniques, para-cliniques, anatomo-pathologiques et thérapeutiques ont été étudiés.

## Résultats

**Figure 1:**
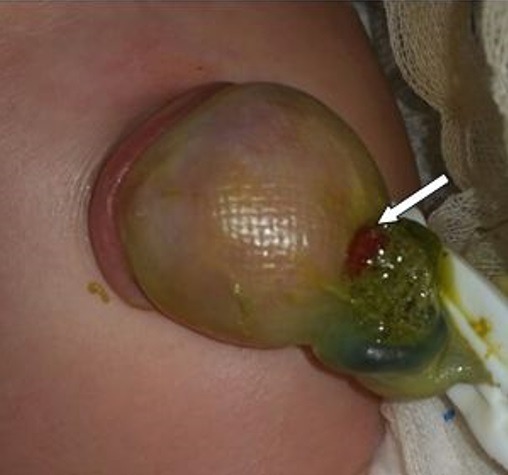
Fistule méconiale sur sac d’omphalocèle [12]

**Figure 2:**
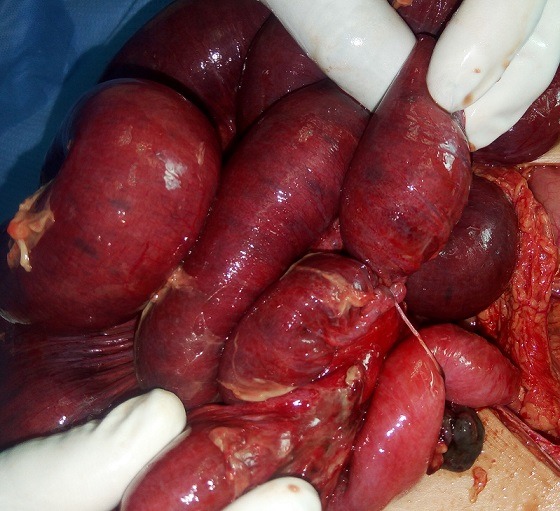
Bride constrictive allant du diverticule de Meckel au mésentère

**Figure 3:**
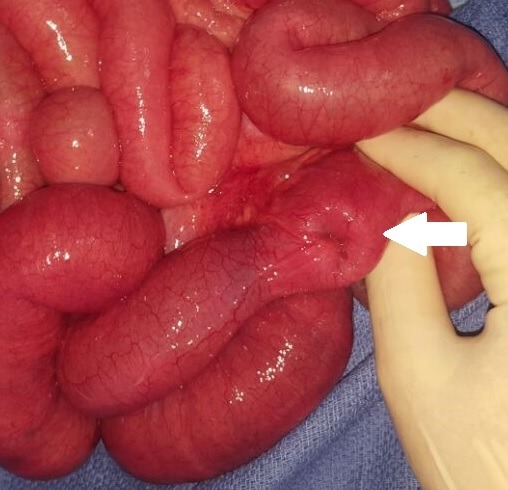
Diverticule de Meckel invaginé dans la lumière de l’anse portante

**Figure 4:**
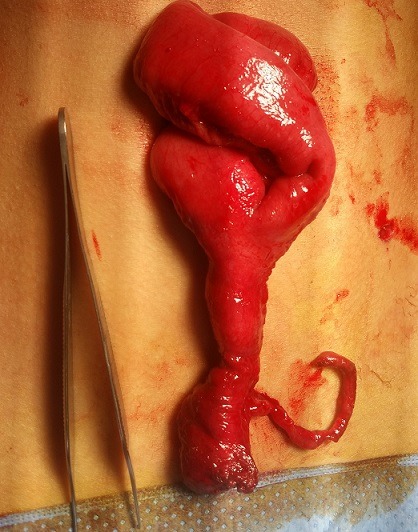
Diverticule de Meckel et anse portante extériorisés par l’incision ombilicale de laparoscopie (out-laparoscopie)

Durant la période de l'étude, 18 cas de DM compliqués (15 garçons et 3 filles) ont été pris en charge dans notre formation. L'âge des patients variait de 1 jour à 15 ans (âge moyen de 5 ans), avec 2 nouveaux nés, 7 nourrissons (entre 1 mois et 2 ans) et 9 enfants âgés de plus de 4 ans. Le premier nouveau né a été admis pour occlusion néonatale basse et le second pour une fistule méconiale sur sac d'omphalocèle (Figure 1). Cinq nourrissons ont été admis pour tableau d'invagination intestinale aigüe (IIA), deux pour syndrome occlusif, et un pour rectorragie de moyenne abondance. Pour les enfants de plus de 4 ans, un cas a été admis pour tableau d'IIA, cinq enfants pour un syndrome occlusif, les 3 autres cas ont présenté respectivement un syndrome appendiculaire, un tableau de péritonite et une rectorragie. L'antécédent de rectorragie de faible abondance a été retrouvé dans un seul cas âgé de 15 ans, admis pour syndrome occlusif. La radiographie de l'abdomen sans préparation (ASP) réalisée devant le tableau d'IIA, de syndrome occlusif ou d'abdomen aigu a montré des niveaux hydro-aériques (NHA) de type grêlique dans 14 cas et une agglutination des anses au niveau de la fosse iliaque droite dans le cas admis pour syndrome appendiculaire. L'échographie abdominale a été réalisée chez tous les patients sauf les 02 nouveaux nés. Elle a objectivé une IIA dans 8 cas (Iléo-colique chez 6 malades et Iléo-iléale chez 2 autres) associée à un épanchement péritonéal de moyenne abondance dans 3 cas. Un épanchement abdominal de moyenne abondance isolé a été retrouvé dans deux cas et une distension des anses grêliques dans 3 cas. Chez le patient admis pour syndrome appendiculaire, l'échographie a montré un épaississement de la dernière anse iléale. Par ailleurs, elle était normale chez les 2 patients présentant une rectorragie. Le scanner abdominal a été réalisé dans 9 cas. Il a confirmé l'IIA iléo-iléale diagnostiquée par échographie chez 3 enfants ayant plus de 2 ans, sans objectiver une cause secondaire de l'invagination. Il a montré une distension grêlique en amant d'un épaississement de quelques anses iléales dans 2 cas et en amant d'une bride primitive dans un autre. Chez le patient admis pour syndrome appendiculaire, le scanner a objectivé un épaississement de la paroi appendiculaire. Par ailleurs il était normal dans les 2 cas ayant une rectorragie. Chez ces deux patients, la fibroscopie oeso-gastro-duodénale et la colonoscopie n'ont pas montré d'anomalies. Une scintigraphie au technétium a été réalisée chez ces deux patients, ayant suspecté le diagnostic dans un cas. Sur le plan thérapeutique, une laparoscopie a été effectuée pour les deux patients ayant une rectorragie et pour l'enfant admis pour syndrome appendiculaire. Une excision du sac a été réalisée chez le nouveau né admis pour fistule méconiale sur omphalocèle. Les autres patients ont été opérés par laparotomie. Dans 6 cas, l'exploration a objectivé une bride constrictive tendue du DM à l'ombilic (3 cas) ou au mésentère (3 cas) (Figure 2), avec nécrose des 4 dernières anses dans un cas et nécrose avec perforation de l'anse portante du DM dans un autre. L'invagination intestinale iléo-colique a été retrouvée chez 5 enfants avec 3 cas de nécrose intestinale, la forme iléo-iléale chez 2 enfants avec un cas de nécrose et une invagination du DM dans la lumière de l'anse portante dans un cas (Figure 3). Une diverticulite a été objectivée chez l'enfant admis pour syndrome appendiculaire et le DM a été objectivé chez les 2 enfants admis pour rectorragie. Par ailleurs, une fistule du DM a été retrouvée dans le cas d'omphalocèle. Une résection segmentaire de l'anse portante du DM et des anses nécrosées suivie d'une anastomose immédiate a été réalisée dans 17 cas dont 3 par out-laparoscopie à travers l'incision ombilicale (Figure 4). L'iléostomie a été réalisée dans un cas, suivie d'un rétablissement de continuité 3 semaines après. Un lâchage d'anastomose a été marqué chez un nouveau né chez qui une iléostomie a été réalisée, suivie d'un rétablissement de continuité après 3 semaines. Par ailleurs l'évolution était bonne pour tous les patients. Sur le plan anatomo-pathologique, deux cas d'hétérotopie ont été notés, dont une gastrique et l'autre pancréatique.

## Discussion

L'incidence du DM dans la population générale a été estimée à 2% [1]. L'incidence des formes symptomatiques de l'enfant est difficile à élucider vu que la plupart des articles publiés sont des séries limitées. Une étude épidémiologique large réalisée par Alemayehu *et al.* a trouvé une incidence de 6,4% [8]. Le DM est plus fréquent chez les garçons, le sex-ratio est de 1,5 à 4/1 [3]. Dans notre série la prédominance masculine est nette (sex-ratio 5/1). Il est bien connu que le DM se complique avant l'âge de 2 ans [9]. Alemayehu [8] et Ruscher [10] ont trouvé que la moitié des enfants opérés pour une complication du DM sont âgés de moins de 5 ans. Dans notre étude 50% des enfants sont âgés de moins de 2 ans. Cependant, les formes à révélation néonatales sont extrêmement rares [11] et les cas rapportés dans la littérature ne concernent que des cas isolés ou des séries limitées. Les deux cas de notre série ont été sujets de publications précédentes [11, 12]. Les complications rapportées sont multiples mais les plus fréquentes sont hémorragiques, obstructives ou inflammatoires [3]. De ce fait, les manifestations varient considérablement en fonction des scénarios cliniques [13, 14]. D'autres formes plus rares ont été décrite comme la hernie de Littré étranglée, les torsions du DM et les tumeurs [4, 15]. Les complications occlusives du DM sont de mécanismes variables [5]. L'obstruction peut être due à une IIA, le DM pourrait altérer le rythme du péristaltisme et être le point de départ de l'invagination [13] surtout par les diverticules courts à base d'implantation large ou inflammés (diverticulite) [5]. En cas d'IIA survenant chez un enfant de plus de deux ans ou en cas d'invagination iléo-iléale, une cause organique doit être recherchée dont la plus fréquente étant le DM [5]. L'occlusion peut être due également à une bride. En effet, 26% des DM sont attachés à l'ombilic par une bande fibreuse et la persistance de l'artère vitelline peut causer la formation d'une bande méso-diverticulaire qui peuvent causer l'occlusion intestinale par strangulation des anses, hernie interne ou volvulus [16]. Dans notre série les complications obstructives sont les plus fréquentes (70%) des cas (6 brides, 7 IIA et une invagination intra-luminale). Le DM peut également s'invaginer en intraluminale et provoquer une obstruction intestinale [17]. Concernant les manifestations inflammatoires, elles sont souvent trompeuses, ainsi, une diverticulite peut être confondue avec une appendicite et le diagnostic ne se fait qu'en per-opératoire [18]. Les complications hémorragiques sont favorisées par la présence d'hétérotopie au niveau du DM, notamment de type gastrique produisant l'acide chlorhydrique et induisant des ulcérations avec érosion des vaisseaux [19]. Elles se manifestent souvent par des rectorragies minimes ou parfois de grande abondance [3]. Les hétérotopies sont trouvés dans 4,6 à 71% des DM symptomatiques [3]. Elles sont le plus souvent gastriques, mais peuvent également être pancréatiques, jéjunales ou coliques [13].

Les présentations cliniques des complications du DM sont variables et les méthodes diagnostiques ne sont pas spécifiques [20]. Les moyens d'imagerie comme la radiographie standard, l'échographie, la tomodensitométrie (TDM), la scintigraphie au Tc-99 peut produire de faux positifs ou faux négatifs [13, 20]. Sur l'échographie ou la TDM le DM prend la forme d'un kyste une d'un sac borgne divergent de l'iléon. Mais, il est difficile de distinguer le DM des boucles adjacentes dans l'intestin grêle. Cependant, la présence d'une bande attachée reliant le DM à l' ombilic ou au mésentère offre une aide supplémentaire pour le diagnostic [21, 22]. La scintigraphie au Tc-99m Pertechnetate peut visualiser le DM, par fixation du traceur dans le tissu ectopique. La sensibilité de cet examen varie de 60 à 90 % et sa spécificité varie de 90% à 98% [21]. Il a joué un rôle important dans le diagnostic étiologique des hémorragies intestinales basses [23]. Malgré ces moyens d'imagerie, le DM est souvent découvert lors de l'exploration chirurgicale. Actuellement la laparoscopie permet le diagnostic et le traitement du DM, notamment chez les enfants avec manifestations hémorragiques ou inflammatoires [22]. Mais en cas de manifestations obstructives, par IIA ou occlusion sur bride, les enfants sont souvent opérés en urgence et le diagnostic du DM se fait par laparotomie [16, 24]. Dans notre série, la laparoscopie a été réalisée pour les deux cas admis pour rectorragie et pour le cas ayant un tableau d'appendicite. Dans la série de Huang *et al*. [9] un tiers des patients ont été opérés par laparoscopie, ceci est similaire aux taux trouvés dans une étude large réalisée aux états unis [10]. Plusieurs techniques de résection de DM ont été décrites comme la résection laparoscopique avec suture intracorporelle ou par agrafeuse linéaire endoscopique, résection-anastomose assistée par laparoscopie ou suture extracorporelle « out laparoscopie » [24, 25]. Dans la résection assistée par laparoscopie trans-ombilicale (out-laparoscopie), le DM et l'anse portante sont extériorisés par l'incision trans-ombilicale et l'anastomose se fait à la main, évitant ainsi la contamination abdominale et l´omission de muqueuse ectopique. Elle est considérée comme sûr et efficace dans la gestion du DM et il est recommandé par la plupart des chirurgiens pédiatres [24, 26].

## Conclusion

Le DM peut rester longtemps asymptomatique mais peut donner lieu à des complications mettant potentiellement en jeu le pronostic vital. Ces complications sont diverses avec des tableaux variables et d'autant plus fréquentes que l'enfant est jeune. Les examens complémentaires sont peu spécifiques et le diagnostic se fait souvent en peropératoire. Ainsi, le DM compliqué doit faire partie des diagnostics à évoquer devant un abdomen aigu, une occlusion intestinale ou hémorragie digestive basse. La laparoscopie diagnostique et thérapeutique est la méthode de choix notamment en cas de complications hémorragiques ou inflammatoires.

### État des connaissances actuelles sur le sujet

- Diverticule de Meckel: malformation fréquente du tube digestif;
- Complications rares.

### Contribution de notre étude à la connaissance

- Complications diverses avec tableaux cliniques variables;
- Diagnostic souvent en per-opératoire;
- Formes particulières des nouveau-nés.

## Conflits des intérêts

Les auteurs ne déclarent aucun conflit d'intérêts.

## References

[1] Lin X-K, Huang X-Z, Bao X-Z, Zheng N, Xia Q-Z, Chen C (2017). Clinical characteristics of Meckel diverticulum in children: a retrospective review of a 15-year single-center experience. Medicine (Baltimore).

[2] Chan KWE, Lee KH, Wong HYV, Tsui SYB, Wong YS, Pang KYK (2014). Laparoscopic excision of Meckel's diverticulum in children: What is the current evidence?. World J Gastroenterol.

[3] Hansen C-C, Søreide K (2018). Systematic review of epidemiology, presentation, and management of Meckel's diverticulum in the 21stst century. Medicine (Baltimore).

[4] Tekou H, Akakpo-Numado Gk, Gnassingbe K, Tchama R, Attipou K (2007). Les diverticules de Meckel chez l'enfant: à propos de 11 cas. Gastroenterol Clin Biol.

[5] Khemekhem R, Ben Ahmed Y, Rahay H, Soufiane G, Said J, Douira W (2013). Les aspects pathologiques du diverticule de Meckel chez l'enfant. Journal de Pédiatrie et de Puériculture.

[6] Diop A, Thiam O, Guèye ML, Seck M, Touré AO, Cissé M (2018). Diverticules de Meckel compliqués: à propos de 15 cas. Pan African Medical Journal.

[7] Méndez-García C, Suárez-Grau JM, Rubio-Chaves C, Martín-Cartes JA, Docobo-Durántez F, Padillo-Ruiz J (2011). Surgical pathology associated with Meckel´s diverticulum in a tertiary hospital: 12 year review. Rev Esp Enferm Dig.

[8] Alemayehu H, Hall M, Desai AA, St Peter SD, Snyder CL (2014). Demographic disparities of children presenting with symptomatic Meckel's diverticulum in children's hospitals. Pediatr Surg Int.

[9] Huang C-C, Lai M-W, Hwang F-M, Yeh Y-C, Chen S-Y, Kong M-S (2014). Diverse presentations in pediatric Meckel's diverticulum: a review of 100 cases. Pediatr Neonatol.

[10] Ruscher KA, Fisher JN, Hughes CD, Neff S, Lerer TJ, Hight DW (2011). National trends in the surgical management of Meckel's diverticulum. J Pediatr Surg.

[11] Oukhouya MA, Andaloussi S, Abdellaoui H, Tazi M, Mahmoudi A, Elmadi A (2018). Meckel's diverticulum causing intestinal obstruction in the newborn. Pan African Medical Journal.

[12] Tazi Charki M, Abdellaoui H, Andaloussi S, Oukhouya MA, Mahmoudi A, El madi A (2019). Congenital fistulisation of Meckel's diverticulum in omphalocele sac: case report. Pan African Medical Journal.

[13] Chen Q, Gao Z, Zhang L, Zhang Y, Pan T, Cai D (2018). Multifaceted behavior of Meckel's diverticulum in children. J Pediatr Surg.

[14] Kotecha M, Bellah R, Pena AH, Jaimes C, Mattei P (2012). Multimodality imaging manifestations of the Meckel diverticulum in children. Pediatr Radiol.

[15] Noukpozounkou SB, Lawani I, Elegbede OTA, Seto DM, Assan BR, Houegban ASCR (2018). Hernie de littré ombilicale étranglée chez l'enfant: complication rare d'une malformation fréquente de l'intestin grêle. Pan African Medical Journal.

[16] Blevrakis E, Partalis N, Seremeti C, Sakellaris G (2011). Meckel's diverticulum in paediatric practice on Crete (Greece): a 10-year review. Afr J Paediatr Surg.

[17] Francis A, Kantarovich D, Khoshnam N, Alazraki AL, Patel B, Shehata BM (2016). Pediatric Meckel's diverticulum: report of 208 cases and review of the literature. Fetal Pediatr Pathol.

[18] Palanivelu C, Rangarajan M, Senthilkumar R, Madankumar MV (2008). Laparoscopic management of symptomatic Meckel's diverticula: a simple tangential stapler excision. Journal of the Society of Laparoendscopic Surgeons (JSLS).

[19] Stănescu GL, Pleşea IE, Diaconu R, Gheonea C, Sabetay C, Şîştea D (2014). Meckel's diverticulum in children, clinical and pathological aspects. Rom J Morphol Embryol.

[20] Menezes M, Tareen F, Saeed A, Khan N, Puri P (2008). Symptomatic Meckel's diverticulum in children: a 16-year review. Pediatr Surg Int.

[21] Baldisserotto M, Maffazzoni DR, Dora MD (2003). Sonographic findings of Meckel's diverticulitis in children. AJR Am J Roentgenol.

[22] Bennett GL, Birnbaum BA, Balthazar EJ (2004). CT of Meckel's diverticulitis in 11 patients. AJR Am J Roentgenol.

[23] Mariani G, Pauwels EKJ, AlSharif A, Marchi S, Boni G, Barreca M (2008). Radionuclide evaluation of the lower gastrointestinal tract. J Nucl Med.

[24] Shalaby RY, Soliman SM, Fawy M, Samaha A (2005). Laparoscopic management of Meckel's diverticulum in children. J Pediatr Surg.

[25] Rattan KN, Singh J, Dalal P, Rattan A (2016). Meckel's diverticulum in children: our 12-year experience. Afr J Paediatr Surg.

[26] Cobellis G, Torino G, Noviello C, Cruccetti A, Mastroianni L, Amici G (2011). Versatility of one-trocar surgery in children. J Laparoendosc Adv Surg Tech A.

